# Applying diagnosis and pharmacy-based risk models to predict pharmacy use in Aragon, Spain: The impact of a local calibration

**DOI:** 10.1186/1472-6963-10-22

**Published:** 2010-01-21

**Authors:** Amaia Calderón-Larrañaga, Chad Abrams, Beatriz Poblador-Plou, Jonathan P Weiner, Alexandra Prados-Torres

**Affiliations:** 1 Aragon Health Science Institute. 25, Gomez Laguna Ave, Floor 11. Zaragoza 50009, Spain; 2 Johns Hopkins Bloomberg School of Public Health. Health Services Research & Development Centre. 624 N. Broadway, Room 605. Baltimore, MD 21205, USA

## Abstract

**Background:**

In the financing of a national health system, where pharmaceutical spending is one of the main cost containment targets, predicting pharmacy costs for individuals and populations is essential for budget planning and care management. Although most efforts have focused on risk adjustment applying diagnostic data, the reliability of this information source has been questioned in the primary care setting. We sought to assess the usefulness of incorporating pharmacy data into claims-based predictive models (PMs). Developed primarily for the U.S. health care setting, a secondary objective was to evaluate the benefit of a local calibration in order to adapt the PMs to the Spanish health care system.

**Methods:**

The population was drawn from patients within the primary care setting of Aragon, Spain (n = 84,152). Diagnostic, medication and prior cost data were used to develop PMs based on the Johns Hopkins ACG methodology. Model performance was assessed through r-squared statistics and predictive ratios. The capacity to identify future high-cost patients was examined through c-statistic, sensitivity and specificity parameters.

**Results:**

The PMs based on pharmacy data had a higher capacity to predict future pharmacy expenses and to identify potential high-cost patients than the models based on diagnostic data alone and a capacity almost as high as that of the combined diagnosis-pharmacy-based PM. PMs provided considerably better predictions when calibrated to Spanish data.

**Conclusion:**

Understandably, pharmacy spending is more predictable using pharmacy-based risk markers compared with diagnosis-based risk markers. Pharmacy-based PMs can assist plan administrators and medical directors in planning the health budget and identifying high-cost-risk patients amenable to care management programs.

## Background

Pharmaceutical public spending is recognised as one of the main cost containment targets in the financing of national health systems such as that in Spain. However, studies aimed at understanding population patterns of drug consumption, costs and morbidity are uncommon. Recent pharmaceutical reimbursement reforms in Spain have focused on measures oriented towards the industry side, giving less importance to the role of patient need and demand [1]. Patient characteristics are not taken into consideration for pharmacy budget allocation to health centres either, ascribing fundamental weight to prior year costs [2].

By taking into account the risk factors for a covered population, prospective risk adjustment methodologies--commonly referred to as "predictive models" (PMs)--can be helpful for health budget planning and case profiling [3]. These tools can also be used as population-based screens meant to identify enrolees who pose a relatively higher risk of generating large future pharmacy expenditures [4,5]. Such patients would benefit from case management programs that could enhance primary care in order to improve the quality and coordination of services [6-8]. As far as physicians are concerned, PMs provide a means of considering health status when assessing drug expenditure, providing health care providers with incentives to be efficient in exercising pharmaceutical benefits.

In this manuscript, we use Adjusted Clinical Group PMs developed at Johns Hopkins University because of their acknowledged validity and extensive use as a risk adjustment technology [9,10]. To date, health services research in the Spanish context has focused on using diagnoses-based risk assessment variables [3,11]. However, risk assessment models such as the ACG-PMs, which integrate routinely collected pharmacy data, may be more appropriate. Chronic conditions are associated with specific pharmacotherapy protocols and, furthermore, diagnostic data often lack complete documentation [12]. This is due not only to the fact that chronic diseases are frequently not explicitly named in physicians' medical records [13], but also because individual data are frequently fragmented across different health care information systems--primary care, secondary care, emergencies etc.--. In contrast, studies carried out in Canada [14,15] showed that prescription claims represent one of the most accurate means of determining what drugs are dispensed to individuals, because pharmacists almost always dispense the medication that is prescribed and this information is reliably transmitted to the drug claims database.

Last, the application of these tools in a different health system from the one where they were originally developed requires the use of local weights for the analysed risk factors. Thus, the role of a calibration may also be appraised in order to maximise the predictive accuracy of the PMs.

The objective of this paper was first to assess the usefulness of incorporating pharmacy data into our efforts to better understand health care resource utilisation and patients' use of pharmacy resources in particular and, second, to evaluate the benefit of a local calibration of PMs in order to adapt them to the Spanish health care system.

## Methods

### Data Source and Study Sample

Data were obtained from the Electronic Medical Records of patients from six primary care health centres belonging to Aragon's Public Health Care System for the years 2006 (Year-1) and 2007 (Year-2). In order to increase the reliability of the data, health centres were selected according to their experience with the use of Electronic Medical Records, which, in all cases, was longer than three years. The sample was restricted to enrolees seen at least once by a public general practitioner (family doctor or paediatrician) during both Year 1 and Year 2, which resulted in a final sample of 84,152. Among the 84,152 patients 9.4% had no pharmacy expenditure in 2006 and 9.3% had no pharmacy expenditure in 2007. Data were obtained from administrative registries of the Aragon Health Care System after official request and authorization. Personal information was anonymised according to the Spanish Organic Law of Personal data Protection 15/1999. This work is part of a project funded by the Carlos III Health Institute which has been approved by the Ethics Committee of Aragon (CEICA).

The Year-1 inputs for the PMs were patients' gender, their age, all assigned diagnoses and the codes for the drugs they consumed according to the ATC Classification System [16]. Diagnoses were originally coded according to the International Classification of Primary Care (ICPC-2) [17]; the codes were subsequently transformed to correspond with the International Classification of Disease (ICD-9-CM) [18]. We used patients' annual pharmacy expenditures from Year-1 to calculate prior pharmacy cost predictors. Year-2 expenditures were only used as a validation tool, in order to assess the performance of the PMs.

To obtain information on pharmacy charges, data from Electronic Medical Records were crossed with the information in the National Pharmacy Database. Registries were made anonymous with the aim of ensuring the confidentiality of the information. These costs correspond to the retail price of drugs consumed and pharmacy accessories used by patients. Even if drug claims collect prescriptions carried out by both specialised and general practitioners, the majority of these prescriptions are filled by the latter (92.6% in 2006 and 92.7% in 2007, according to the Pharmacy Database of Aragon).

### Risk Factors Within the Predictive Models

Based on the abovementioned input variables, the ACG case-mix system, version 8.1^®^, assigns risk measurement variables related to specific demographic, diagnoses, and pharmacy use patterns.

Diagnosis-based risk factors include Adjusted Clinical Groups implying the highest levels of medical need (ACGs are a series of mutually exclusive health status categories defined by morbidity, age, and sex [9,10]), specific Expanded Diagnostic Clusters representing uncommon diseases that signify high disease severity (EDCs are groupings of ICD-9-CM codes according to clinical similarity [19]), and the hosdom and patient frailty markers (the hosdom marker is a count of different morbidity types associated with a >50% probability of future hospital admission among patients and the frailty marker clusters diagnostic codes that indicate the presence of at least 1 to 11 frail conditions [19]).

As for medication-derived risk factors, several pharmacy-based morbidity groups (Rx-MG) are included. Rx-MGs are created to account for the anatomical-physiological system drugs act on, as well as the morbidity differentiation, the expected duration and the severity of the diseases to be treated using the medication. These four clinical dimensions not only characterise medications by morbidity type but also have major consequences for predictive modelling. Higher levels of differentiation and chronicity, as well as greater severity, would all be expected to increase resource use [19].

Whereas DxRx-PMs include the whole set of risk factors, Dx-PMs and Rx-PMs will only use diagnostic or pharmacy-based variables, respectively.

### Data Analysis

To understand the differences between the U.S. and Spanish health care systems, we first described, for each of the settings, each patient's demographic and clinical characteristics from Year-1 as well as the distribution of the pharmacy expenditures from Year-2. U.S. data, which were originally used for the empirical development of the ACG-based PMs, were obtained from the PharMetrics Patient-Centric Database. This database represents the medical and pharmacy claims and enrolment records across the continuum of medical care for approximately 85 geographically diverse health plans within the U.S.

Pharmacy data were used as a means of assessing the validity of diagnoses recorded in physicians' medical records. Comparisons were made between patients identified as having specific chronic conditions using diagnosis codes (ICD-9-MC), pharmacy codes (ATC Classification System), and both diagnoses and pharmacy codes.

Next, we looked at how useful Rx-PMs were for predicting future pharmacy expenditures. Multivariate linear regression was employed to obtain r-squared statistics by regressing Year-2 pharmacy charges on the PMs' risk factors.

We also calculated predictive ratios (PRs) to assess model accuracy. We used Year-2 as the validation sample and applied models estimated from Year-1 to generate predictions for each patient and PRs for 3 types of cohorts defined by a) the presence of a relevant diagnosis, b) the presence of a relevant drug claim and c) pharmacy costs in Year-1 arrayed by percentiles.

Classification accuracy was examined using logistic regression in which the dependent variable was defined by assignment (or not) to the top 5% risk group in terms of Year-2 charges (high consumer yes/no). Model fit was examined through c-statistic, sensitivity and specificity parameters.

For each of the mentioned measures, results from a local calibration of the model were compared with results based on the U.S. health care system.

Finally, we performed a sensitivity analysis to measure how results would vary when using U.S. weights derived from a Medicare managed care population for individuals over the age of 65. This is an alternative to the commercial reference weights obtained from the PharMetrics Patient-Centric Database, which is available as part of the ACG software for predominantly elderly populations.

## Results

The ACG case-mix system proved to have high performance in recognising and assigning 100% of the diagnoses and 92.5% of the pharmacy codes.

The Spanish and U.S. datasets had similar distribution in terms of age, sex and chronic conditions (Table 1). With regard to the prevalence of cited diseases, numbers were comparable except for depression: compared with what was found in the U.S. data, the proportion of the study sample having this condition was nearly two times higher for the young Spanish population and around four times higher for the elderly Spanish population. In both Spanish and U.S. datasets, the distribution of the pharmacy expenditure showed a pronounced skewness, especially among the under-65 population, where nearly half of annual expenditures on prescription drugs were generated by only 5% of the study population. Regarding the elderly population, U.S. data showed a more skewed distribution, with half of the study population being responsible for almost 90% of pharmacy expenses. These numbers are consistent with the well-known distribution properties of pharmacy costs in a population.

**Table 1:** Characteristics of the Spanish study population and U.S. benchmark population.

	Spanish Data				U.S. Data			
	**Under 65 population**		**Over 65 population**		**Under 65 population**		**Over 65 population**	

**Demographic Characteristics (Year-1)**								

Age (years) %								
0-4	6.8		---		6.6		---	
5-11	7.6		---		11.6		---	
12-17	6.2		---		10.3		---	
18-34	28.8		---		24.5		---	
35-44	16.2		---		19.5		---	
45-54	16.8		---		17.3		---	
55-64	17.6		---		10.2		---	
65-69	---		24.3		---		24.0	
70-74	---		27.3		---		28.9	
75-79	---		22.6		---		22.3	
80-84	---		15.7		---		14.6	
>85	---		10.1		---		10.2	
Age (years) mean	34.5		75.0		31.5		75.4	
Female %	54.9		58.5		51.5		57.8	

**Clinical characteristics (Year-1)**								

Chronic conditions %								
None	59.3		8.3		68.1		13.3	
1	24.4		19.1		17.4		14.1	
2 or more	16.3		72.6		14.5		72.6	
Mean no. of chronic conditions	0.7		2.7		0.6		3.2	
Prevalence of the diseases:								
Hypertension %	9.2		54.8		7.7		53.9	
Hyperlipidaemia %	10.6		32.7		7.3		35.0	
Depression %	6.3		11.9		3.6		3.3	
Diabetes %	3.3		18.4		2.7		18.4	
Asthma %	5.0		4.2		3.7		4.0	
CHF %	0.2		3.8		0.3		7.2	

**Pharmacy expenditure (Year-2)**								

Mean pharmacy expenditure	228 €		950 €		365 € *($467)*		840 € *($1,077)*	
Mean pharmacy expenditure of:		**% of total €**		**% of total €**		**% of total $**		**% of total $**
								
Highest 1%	4,998 €	21.9	6,838 €	7.2	7,608 € *($10,077)*	21.5	11,137 € *($14,764)*	13.7
Highest 5%	2,255 €	49.5	4,064 €	21.4	3,399 € *($4,502)*	48.1	4,677 € *($6,200)*	28.8
Highest 10%	1,518 €	66.7	3,214 €	33.8	2,312 € *($3,062)*	65.4	3,302 € *($4,377)*	40.6
Highest 30%	693 €	91.3	2,083 €	65.8	1,090 € *($1,443)*	92.4	1,922 € *($2,548)*	71.0
Highest 50%	443 €	97.2	1,601 €	84.3	701 € *($928)*	99.0	1,444 € *($1,914)*	88.9

Source of currency rate: XE.com Currency Services as of 23/01/2009.

CHF: Congestive Heart Failure.

**Figure 1:**
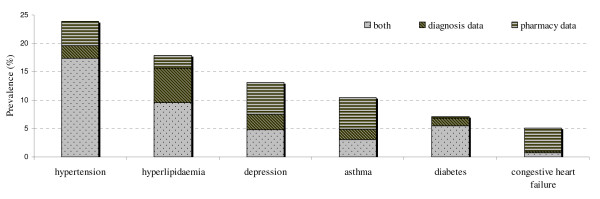
**Prevalence of chronic disease in the general population according to diagnosis and pharmacy data**.

With regard to comparisons between "who" was identified as having a particular medical condition using diagnostic data from electronic medical records and "who" was identified as having a medical condition using pharmacy claims, we found that, for several chronic conditions such as depression or asthma, some patients taking drugs have no diagnoses associated with these drugs. On the other hand, a high percentage of individuals to whom the diagnosis of hyperlipidaemia has been assigned are not taking lipid-lowering drugs according to pharmacy claims (Figure 1).

Across all PMs, the DxRx model explained the most variance (Table 2). However, the capacity of the RxPM to predict future pharmacy expenditures was almost as high as that of the DxRx combined model (40.6% vs. 42.6%). In all cases, models provided considerably better predictions when applying weights resulting from local calibrations.

**Table 2:** Statistical performance of Alternative Predictive Models using U.S./Spanish weights.

	Alternative Predictive Models					
	**Dx-PM**		**Rx-PM**		**DxRx-PM**	

	**U.S. Weights**	**Spanish Weights**	**U.S. Weights**	**Spanish Weights**	**U.S. Weights**	**Spanish Weights**

**Variance explained. R^2^**	18.9%	29.4%	22.2%	40.6%	23.5%	42.6%
Area Under ROC Curve*	.868	.902	.900	.941	.903	.949
Sensitivity*	30.6%	39.4%	27.5%	52.3%	31.2%	53.2%
Specificity*	96.3%	96.8%	96.2%	97.5%	96.4%	97.5%
Mean pharmacy expenditure Year-2 (€)*						
True positives	3,059	3,076	3,244	3,236	3,248	3,244
True negatives	233	233	236	234	235	234

*Outcomes refer to top 5% Year-2 pharmacy cost group. The Area Under ROC Curve ranges from 0.5 (model no better than the flip of a coin) to 1.0 (perfect true positive and true negative classification).

Dx: physician assigned diagnosis. Rx: pharmacy prescriptions filled by physicians. PM: predictive model.

Table 3 shows means and PRs for Year-2 pharmacy expenditures based on information from the prior year. The most expensive of these groups (those who had congestive heart failure, were taking drugs for congestive heart failure or were in the top 1% of spending during Year-1) incurred costs between 4 and 15 times higher than the average of 390€. The diagnosis-based model (Dx-PM) predicted pharmacy expenditures more accurately for the diagnosis-identified groups, but somewhat underpredicted the groups defined by their use of drugs. Analogously, the drug-based model (Rx-PM) was more accurate with the groups defined by their use of drugs in comparison with the groups defined by medical conditions. The benefit of a local calibration was persistent in all cases.

**Table 3:** Predictive Ratios for Year-2 pharmacy costs for Disease, Drug Use and Cost Defined Groups.

			Alternative Predictive Models					
			**Dx-PM**		**Rx-PM**		**DxRx-PM**	

	**n**	**Mean pharmacy expenditure Year-2 (€)**	**U.S**. **Weights**	**Spanish** **Weights**	**U.S**. **Weights**	**Spanish** **Weights**	**U.S**. **Weights**	**Spanish** **Weights**

**Medical condition groups**								

Hypertension	16,519	931	0.773	0.999	0.856	0.989	0.864	1.000
Hyperlipidaemia	13,175	811	0.776	0.925	0.900	1.007	0.911	1.010
Depression	6,326	876	0.847	1.000	0.961	0.979	1.009	1.000
Diabetes	5,724	1,180	0.714	1.000	0.774	0.984	0.793	1.000
Asthma	4,090	522	0.951	1.000	0.921	0.959	0.986	1.000
CHF	864	1,396	1.031	1.000	0.755	0.958	0.774	1.000

**Drug utilisation groups**								

Antihypertensives	18,209	990	0.721	0.918	0.856	1.000	0.859	1.000
Lipid-lowering	9,912	1,084	0.638	0.811	0.843	1.000	0.853	1.000
Antidepressants	8,717	1,023	0.646	0.774	0.920	1.000	0.936	1.000
Antidiabetics	4,947	1,267	0.654	0.917	0.785	0.998	0.797	1.002
Antiasthmatics	7,303	755	0.716	0.820	0.870	1.000	0.874	1.000
CHF	3,981	1,349	0.687	0.822	0.773	1.000	0.774	1.000

**Year-1 spending percentiles**								

Highest 1%	841	5,708	0.175	0.240	0.247	0.345	0.249	0.368
Highest 5%	4,207	3,026	0.301	0.421	0.419	0.548	0.421	0.572
Highest 10%	8,415	2,221	0.378	0.519	0.517	0.642	0.519	0.665
Highest 30%	25,245	1,168	0.582	0.736	0.735	0.835	0.738	0.846
Highest 50%	42,075	766	0.746	0.859	0.882	0.935	0.886	0.938

Predictive ratios reflect the ratio of expected pharmacy cost divided by the actual cost for each cohort (PR = 1 indicates perfect prediction, PR<1 indicates underprediction and PR>1 indicates overprediction). Medical condition groups consist of patients with at least 1 relevant diagnosis in Year-1; drug utilisation groups include those with at least one relevant pharmacy fill in Year-1.

Dx: physician assigned diagnosis. Rx: pharmacy prescriptions filled by physicians. PM: predictive model. CHF: Congestive Heart Failure.

Table 2 also shows the area under ROC curve and sensitivity and specificity values of each of the models evaluated for the top 5% Year-2 cost group. Whereas the 3 models exhibited similar performance on the specificity test, performance on sensitivity diverged: the DxRx-PM and the Rx-PM using Spanish weights showed the best performance (53.2% and 52.3% respectively). This is confirmed by the corresponding ROC curve values (0.949 and 0.941 respectively). Once again, there was little improvement in sensitivity or ROC curve values for the Rx-PM when adding the diagnostic information with regard to the prediction of pharmacy expenditure. The results were notably enhanced when Spanish weights were applied.

## Discussion

No previously published research assesses the usefulness of incorporating pharmacy data into prospective risk adjustment techniques in any national health system. To date, research in the European and Spanish contexts has focused on using diagnoses-based risk assessment variables [3,11]. However, our study, as well as others carried out within the Spanish National Health System [20], determined that the accuracy of the diagnostic codes allocated by primary health care physicians in their computerised medical records could be improved.

The results of this study confirm that much can be learned by looking at pharmacy data, especially when forecasting drug expenditures. Studies carried out in the U.S [21,22] and Europe [23] have outlined the potential of pharmacy data to improve the system of risk adjustment for both care management program capitation payments and pharmacy budget planning. This is of particular interest in a situation in which the information related to drug consumption is routinely recorded and automated, as it is in Spain. Moreover, the fact that the applied drug classification system (ATC classification) is uniform and used all over the country makes the use of pharmacy data very feasible--even more so if we consider the regular updating of the national ATC code databases issued by the Ministry of Health, according to the Guidelines of the World Health Organisation [24].

Nevertheless, several challenges are posed by medication-only PMs. First, using pharmacy data as a risk adjuster for resource allocation could create perverse incentives, encouraging inappropriately prescribed drugs to be given higher budgets and promoting the inappropriate use of these drugs in the future. In consequence, if pharmacy data are to be used for budget allocation purposes, intensive monitoring activity will be required to prevent the inappropriate prescribing of drugs. As it happens in other European countries [23], in Spain, social security funds are not care providers themselves unlike Health Maintenance Organizations (HMO) in the U.S.; this may reduce the danger of inappropriate prescription behaviour.

Second, pharmacy claims data may not always portray an accurate clinical picture, because some prescribed medications have multiple indications from which a patient's disease status must be inferred and because one disease may have many medication options in terms of its management [25]. One of the most important innovations presented by the Rx-PM from the ACG system is its clinically oriented approach, which captures the unique clinical information embedded in medication-use data instead of attempting to identify diseases/conditions based on medications [19].

The benefits of local calibrations have become evident. As was the case with other risk adjustment tools, such as the Diagnosis-Related Groups used in acute care hospitals, adaptation processes have been developed by other countries on the grounds that the philosophy of health care, resource consumption patterns and funding approaches differ from those in the country where the tool was originally created [26]. Indeed, importing clinical predictors related to weights resulting from empirical evaluations carried out with U.S. cost data could lead to the incorporation of U.S. funding incentives and disincentives into the Spanish health care system. The results of our work show that the statistical performance of PMs was optimised using Spanish weights. This is due to the fact that local weights were calculated by regressing pharmacy cost data from our own health system on the explanatory variables for ACG-PMs.

Moreover, our sensitivity analysis demonstrated that these results were fairly robust even when using U.S. weights derived from a Medicare managed care population for individuals over the age of 65.

### Limitations

The main limitation of this study is related to the infeasibility of applying a split half method, which involves dividing the total sample in two and correlating the results, as a way of assessing the reliability of a test. In order to apply this technique to our particular study, the sample size would need to be larger than 80,000 patients so that each of the split groups would surpass the minimum number of individuals required for predictive modelling. On the other hand, the study population is not representative of either the population of the Aragon region or that of Spain, so results must be cautiously interpreted. Moreover, given the applied patient inclusion criteria (i.e. enrolees seen at least once by a public general practitioner during both Year 1 and Year 2), 7.9% of the enrolees that went to the health centre in 2006 (Year-1) and 14.3% of the enrolees that went in 2007 (Year-2) were excluded from the study sample due to their non-attendance in 2007 and 2006 respectively. Future studies may consider including patients with a discontinuous utilization of health care services when measuring the performance of PMs.

Nevertheless, the validity of the sample is backed up by the fact that the proportion of women, the age distribution, the prevalence of chronic conditions and the behaviour of patients with regard to pharmacy expenditure are consistent with those of previous studies carried out in the Spanish primary care setting [3,11].

Another potential limiting factor could be related to the relatively recent incorporation of electronic medical records into the primary care setting. Even if a series of inclusion criteria were applied during the health centre selection process to guarantee the quality and reliability of the clinical data, a three-year period of experience in the use of electronic records is still short enough that we might question the maturity of the information systems. This could lead to an overestimation of the clinical under-coding effect highlighted in this study. Thus, using even slightly more recent data could substantially boost model performance measures.

An additional reason for overestimating the under-coding phenomenon arises from the lack of connection between databases for primary and specialised care. Although the primary care general practitioners are considered the gatekeepers of the health system and would therefore need to have a recompilation of all diagnostic episodes of a patient, certain codes could be missing when these disease are followed by specialised physicians. In the study region, specialised physicians have poorer information systems than primary care physicians and, consequently, data are less available. Still, drug claims collect prescriptions carried out by both specialised and general practitioners. This situation could explain cases in which patients consume drugs for diseases that are not registered in general practitioners' office records, as reported in this paper.

Even if these two previous statements have been considered as potential limitations of the study due to their association with a poor quality of the data, they support the need to incorporate pharmacy data when carrying out risk adjustment.

### Implications for the Spanish National Health System

When the target of a health care organisation is the management of an individual's medication use, predictive models based on pharmacy data are particularly useful. Adding diagnostic markers to medication data does not appear to improve predictions for pharmacy costs [21,22], which tend to show a pronounced degree of persistence from year to year, particularly among the heaviest users [27]. This has long been the rationale for using prior costs in themselves for budget planning by hospitals and the primary care setting in Spain [2]. In terms of statistical performance, prior cost is a fairly good predictor of future cost--even better than diagnostic or pharmacy-related variables [3]--, but it has some limitations. First, prior cost has no inherent clinical meaning, and is therefore of low relevance to clinicians who wish to intervene. It is not tied to morbidity and, thereby, cannot be translated into clinical action. Second, prior cost is subject to the phenomenon of regression to the mean (i.e., the natural tendency of groups of individuals who are high cost one year to move towards mean costs in the following years). Third, prior-use measures are not entirely appropriate as risk factors for risk-adjusted rate setting or profiling as they potentially could provide incentives to excessive and inappropriate pharmacy use.

Screening tools based on diagnostic or medication data can identify reliable "early warning signs" of future expenses that can then promote secondary prevention through patient care management [4]. Although the beneficial effects of care management have not been consistently demonstrated[28], preliminary evidence from an intensive nurse-based intervention for high-risk elderly individuals appears to show that it holds great promise in terms of cost reduction[29] and better quality of care [30]. A randomised clinical trial carried out recently in the Spanish primary care setting has confirmed the effectiveness of intervention strategies in decreasing the number of consultations of frequent attenders[31], paving the way for the implementation of further cost efficiency-focused strategies.

Moreover, the optimal predictive capacity of the ACG-PM proves its usefulness for future budget planning. This has been demonstrated to entail the largest impact on pharmaceutical revenues among existing pharmacy regulatory measures[32].

Last, PMs provide a means of determining physician prescription profiles while adjusting for patient case-mix, so decisions about incentives, efficiency improvement efforts or even sanctions can be targeted towards the "right" physicians [33].

## Conclusion

Pharmacy-based PMs can assist plan administrators and medical directors in planning the health budget and identifying high-cost-risk patients amenable to care management programs.

An application focussing on the total expenditure of the primary care setting is the next research approach required. While pharmacy spending is particularly predictable from drug data, diagnoses may be more useful than drugs for predicting other medical costs and total costs [4,22,34]. There could also be factors, such as behavioural risks or functional health status, that risk models do not yet capture well, and whose influence on pharmacy consumption ought to be considered in future studies.

## Competing interests

The ACG System is commercially available under license with The Johns Hopkins University, which holds the copyright. Johns Hopkins University benefits financially from the sale of this software. A portion of these royalties is used to support ongoing development work on the system, including the research presented within this article.

## Authors' contributions

ACL, CA, JPW and APT, generated the research question. ACL and BPP carried out the statistical analysis. ACL, CA, JPW and APT participated in the interpretation and discussion of the results. ACL, CA and APT contributed to the drafting of the paper. ACL coordinated the writing of the article. All authors have read and approved the final manuscript.

## Pre-publication history

The pre-publication history for this paper can be accessed here:

http://www.biomedcentral.com/1472-6963/10/22/prepub

## References

[B1] Puig-Junoy J Incentives and pharmaceutical reimbursement reforms in Spain Health Policy 2004 67 149 165 10.1016/S0168-8510(03)00113-1 14720633

[B2] Garcia-Sempere A Peiro S [Drug expenditure in primary care: associated variables and allocation of drug budgets according to health district] Gac Sanit 2001 15 32 40 11333623 10.1016/s0213-9111(01)71515-4

[B3] Garcia-Goni M Ibern P Predictability of drug expenditures: an application using morbidity data Health Econ 2008 17 119 126 10.1002/hec.1238 17427265

[B4] Meenan RT Goodman MJ Fishman PA Hornbrook MC O'Keeffe-Rosetti MC Bachman DJ Using risk-adjustment models to identify high-cost risks Med Care 2003 41 1301 1312 10.1097/01.MLR.0000094480.13057.75 14583693

[B5] Weir S Aweh G Clark RE Case selection for a Medicaid chronic care management program Health Care Financ Rev 2008 30 61 74 19040174 PMC4195045

[B6] Boyd CM Boult C Shadmi E Leff B Brager R Dunbar L Guided care for multimorbid older adults Gerontologist 2007 47 697 704 17989412 10.1093/geront/47.5.697

[B7] Counsell SR Callahan CM Clark DO Tu W Buttar AB Stump TE Geriatric care management for low-income seniors: a randomized controlled trial JAMA 2007 298 2623 2633 10.1001/jama.298.22.2623 18073358

[B8] Morisky DE Kominski GF Afifi AA Kotlerman JB The Effects of a Disease Management Program on Self-Reported Health Behaviors and Health Outcomes: Evidence From the "Florida: A Healthy State (FAHS)" Medicaid Program Health Educ Behav 2009 36 3 505 17 10.1177/1090198107311279 18292218

[B9] Starfield B Weiner J Mumford L Steinwachs D Ambulatory care groups: a categorization of diagnoses for research and management Health Serv Res 1991 26 53 74 1901841 PMC1069810

[B10] Weiner JP Starfield BH Steinwachs DM Mumford LM Development and application of a population-oriented measure of ambulatory care case-mix Med Care 1991 29 452 472 10.1097/00005650-199105000-00006 1902278

[B11] Sicras-Mainar A Serrat-Tarres J [Measurement of relative cost weights as an effect of the retrospective application of adjusted clinical groups in primary care] Gac Sanit 2006 20 132 141 10.1157/13087324 16753090

[B12] Fishman PA Goodman MJ Hornbrook MC Meenan RT Bachman DJ O'Keeffe Rosetti MC Risk adjustment using automated ambulatory pharmacy data: the RxRisk model Med Care 2003 41 84 99 10.1097/00005650-200301000-00011 12544546

[B13] Fowles JB Lawthers AG Weiner JP Garnick DW Petrie DS Palmer RH Agreement between physicians' office records and Medicare Part B claims data Health Care Financ Rev 1995 16 189 199 10151888 PMC4193526

[B14] Levy AR O'Brien BJ Sellors C Grootendorst P Willison D Coding accuracy of administrative drug claims in the Ontario Drug Benefit database Can J Clin Pharmacol 2003 10 67 71 12879144

[B15] Tamblyn R Lavoie G Petrella L Monette J The use of prescription claims databases in pharmacoepidemiological research: the accuracy and comprehensiveness of the prescription claims database in Quebec J Clin Epidemiol 1995 48 999 1009 10.1016/0895-4356(94)00234-H 7775999

[B16] WHO Collaborating Centre for Drug Statistics Methodology Anatomical Chemical Classification Index with Defined Daily Doses (DDD). Oslo 2008

[B17] Lamberts H Wood M ICPC International Classification of Primary Care 1987 Oxford: Oxford University Press

[B18] Información y Estadísticas Sanitarias Ministerio de Sanidad y Consumo Clasificación Internacional de Enfermedades CIE-9-MC. 9a Revisión Modificación Clínica. Madrid 2008

[B19] The Johns Hopkins ACG® System Reference Manual. Version 8.1. Baltimore 2007

[B20] Orueta JF Urraca J Berraondo I Darpon J [Can primary care physicians use the ICD-9-MC? An evaluation of the quality of diagnosis coding in computerized medical records] Gac Sanit 2006 20 194 201 10.1157/13088850 16756857

[B21] Zhao Y Ash AS Ellis RP Ayanian JZ Pope GC Bowen B Predicting pharmacy costs and other medical costs using diagnoses and drug claims Med Care 2005 43 34 43 15626932

[B22] Forrest CB Lemke KW Bodycombe DP Weiner JP Medication, diagnostic, and cost information as predictors of high-risk patients in need of care management Am J Manag Care 2009 15 41 48 19146363

[B23] Lamers LM Pharmacy costs groups: a risk-adjuster for capitation payments based on the use of prescribed drugs Med Care 1999 37 824 830 10.1097/00005650-199908000-00012 10448725

[B24] Instituto Nacional de la Salud Subdirección General de Asistencia Sanitaria. Área de Gestión de Farmacia: Sistema de codificación de principios activos y Dosis Diarias Definidas del INSALUD. Madrid: 2002

[B25] Powers CA Meyer CM Roebuck MC Vaziri B Predictive modelling of total healthcare costs using pharmacy claims data: a comparison of alternative econometric cost modelling techniques Med Care 2005 43 1065 1072 10.1097/01.mlr.0000182408.54390.00 16224298

[B26] Pilla J Hindle D Adapting DRGs: the British, Canadian and Australian experiences Health Inf Manag 1994 24 87 93 10141151 10.1177/183335839402400304

[B27] Coulson NE Stuart B Persistence in the use of pharmaceuticals by the elderly. Evidence from annual claims J Health Econ 1992 11 315 328 10.1016/0167-6296(92)90006-M 10122541

[B28] Luck J Parkerton P Hagigi F What is the business case for improving care for patients with complex conditions? J Gen Intern Med 2007 22 3 396 402 10.1007/s11606-007-0293-2 18026808 PMC2150614

[B29] Sylvia ML Griswold M Dunbar L Boyd CM Park M Boult C Guided care: cost and utilization outcomes in a pilot study Dis Manag 2008 11 29 36 10.1089/dis.2008.111723 18279112

[B30] Boyd CM Shadmi E Conwell LJ Griswold M Leff B Brager R A pilot test of the effect of guided care on the quality of primary care experiences for multimorbid older adults J Gen Intern Med 2008 23 536 542 10.1007/s11606-008-0529-9 18266045 PMC2324149

[B31] Bellon JA Rodriguez-Bayon A de Dios LJ Torres-Gonzalez F Successful GP intervention with frequent attenders in primary care: randomised controlled trial Br J Gen Pract 2008 58 324 330 10.3399/bjgp08X280182 18482486 PMC2435670

[B32] Sood N de VH Gutierrez I Lakdawalla DN Goldman DP The effect of regulation on pharmaceutical revenues: experience in nineteen countries Health Aff (Millwood) 2009 28 125 137 10.1377/hlthaff.28.1.w125 PMC3829766 19088100

[B33] Salem-Schatz S Moore G Rucker M Pearson SD The case for case-mix adjustment in practice profiling. When good apples look bad JAMA 1994 272 871 874 10.1001/jama.272.11.871 8078165

[B34] Sales AE Liu CF Sloan KL Malkin J Fishman PA Rosen AK Predicting costs of care using a pharmacy-based measure risk adjustment in a veteran population Med Care 2003 41 753 760 10.1097/00005650-200306000-00008 12773841

